# Gender difference of geographic distribution of physicians in Japan: three-point analysis of 1994, 2004 and 2014

**DOI:** 10.1186/s12913-023-10258-4

**Published:** 2023-12-13

**Authors:** Kazuki Kimura, Kazuo Inoue, Takahito Ando, Masanori Ito

**Affiliations:** 1 Department of General Internal Medicine, Hiroshima University Hospital, Hiroshima City, Japan 1-2-3 Kasumi, Minami-ku, Hiroshima Prefecture; 2 Department of Community Medicine, Chiba Medical Center, Teikyo University School of Medicine, Chiba, Japan; 3 Laboratory of Pharmaceutical Care and Community Medicine, Faculty of Pharma-Sciences, Teikyo University, Tokyo, Japan

**Keywords:** Physician, Gender difference, Geographic distribution, Year of experience, Japan

## Abstract

**Background:**

Japan's medical education system produces 9,000 graduates annually. Despite the government's implementation of several strategies, including increasing the number of doctors trained, the country still struggles with a shortage of physicians in rural areas. This study examined this issue, focusing on gender and considering years of physician experience, demographic and geographic factors.

**Methods:**

We analyzed the Physician Census from 1994, 2004, and 2014, examining data on physicians’ gender and the number of years since licensure. To correct the impact of municipal mergers, the analysis was aligned with the number of municipalities in 2014 (1741). We examined data from each physician (gender and years of medical experience) and analyzed the demographic and geographic distribution trend using Spearman correlation coefficients. We then used the Gini coefficient to evaluate the distribution change of physicians based on gender and years of experience.

**Results:**

The number of physicians increased 1.29-fold over the 20-year observation period (1.23-fold for male physicians and 2.17-fold for female physicians), and the percentage of female physicians increased from 13.4% to 20.4%. We found that 87.7% of physicians were concentrated in the top 1/3 municipalities in terms of population. The number of female physicians was higher at 91.8% compared to 86.8% for male physicians. The Gini coefficients were lower for veteran physicians of both sexes than for younger physicians. The Gini coefficient for all physicians was 0.315–0.298–0.298 (male physicians: 0.311–0.289–0.283, female physicians: 0.394–0.385–0.395) The Gini coefficients for female compared to male physicians were higher in all age groups, showing that The distribution of female physicians is skewed toward urban areas.

**Conclusion:**

Female physicians are less distributed in rural areas than male physicians. In addition, despite the fact that the number of female physicians has increased more than male physicians over the past 20 years, the geographic ubiquity of female physicians has not improved. Since the trend of increasing the number of female physicians is expected to continue in the future, it is necessary to take some measures, such as providing a work-life balance suitable for female physicians.

**Supplementary Information:**

The online version contains supplementary material available at 10.1186/s12913-023-10258-4.

## Background

To alleviate a shortage of physicians in the 1970s, during a period of economic growth, the Japanese government adopted the “one-prefecture, one-medical-school” policy, and the number of medical school students increased to 8,000. When students graduated from medical school, they tended to remain in the prefecture where they had studied, and there was an equal distribution of physicians throughout Japan. Because of a predicted birthrate decline and an excess of physicians, the number of medical school places was maintained for the next 30 years [1].

In 2004, a new post-graduate two-year clerkship program was created in which the participants rotated among multiple specialties [2], not always at their home university, which ultimately influenced where physicians were located when they went into practice. No longer did the younger generation continue an affiliation with their home university hospital but rather ended up working at city training hospitals. As a result, the number of young physicians practicing at university hospital medical departments decreased, and the clinical work at university hospitals continued to be covered by mid-career physicians [3]. Although physicians now have more control over their own careers and access to case experience and research, this trend has further accelerated the shortage of physicians in rural areas [4].

Overall, the absolute number of physicians in Japan is considered low; among the Organization for Economic Co-operation and Development (OECD) countries,, Japan has only about 60% of the OECD average of physicians (number of physicians per 100,000 population [5]. The capacity of medical schools has been increased to about 9,000/year, and some medical schools have introduced a regional quota system that requires graduates of medical school to work in rural areas; however, there is still a serious shortage of physicians in underpopulated regions [6]. Numerous papers have been published on the distribution of physicians in Japan, and, until the 2000s, many of them supported the idea that the uneven distribution of physicians throughout the country could be corrected by increasing the total number [7–9]. However, in recent years, there have been some studies that argue the opposite [10–12].

Our earlier study found that the distribution of physicians to rural areas has been limited, despite the increase in the overall number of physicians since 2004 (after the clinical training system was implemented) [13]. Moreover, there has been a clear difference in the distribution of male and female physicians both demographically and geographically, with remote small-population municipalities most often being served by middle-aged male physicians, as demonstrated in some studies [14].

As shown in Table 1, the total number of physicians increased by 80,686 (46,457 males and 34,229 females) in the 20 years from 1994 to 2014. With the entry of female students into medical schools, the percentage of male students has dropped from 87.3% in 1994 to 79.6% in 2014.

**Table 1:** Number of physicians (overall and by gender) in 1994, 2004, and 2014

(Number)	1994		2004		2014	
Total physician	230,519		270,371		311,205	
Variation (rate) from 1994	-		△39,852	117.3%	△80,686	129.2%
Number of 100,000 population	184.4		211.7		244.9	
Male physician / (ratio)	201,244	87.3%	225,743	83.5%	247,701	79.6%
Female physician / (ratio)	29,275	12.7%	44,628	16.5%	63,504	20.4%

While the increase in the percentage of female physicians may have an impact on the distribution of physicians, Japan still had the lowest percentage of female physicians among OECD countries as of 1994 [5]. Table 1 shows net gain of physicians, female physicians accounted for 38% in 1994–2004 and 46% in 2004–2014. Some medical schools have a 50/50 male/female ratio in the most recent intake [15]. In addition, there is (only one) female medical college in Japan. From this background, the ratio of female physician tends to increase. As of 2014, the last year of this study, the percentage of female physicians was 20.4%, and it will eventually rise to 30%–35% if this trend continues.

Studies of physician distribution by gender have been conducted overseas, but research in Japan is still in its infancy. According to overseas studies, some literature points out that female physicians have difficulty in obtaining a medical specialty due to life events (marriage, pregnancy, childbirth, childcare, etc.) [16]. Female physicians may be forced to give up studying for a physician career equal to that of their male counterparts due to work-life balance concerns [17, 18]. Remote area medicine often requires a generalist approach to treating a wide range of diseases, rather than a specialist approach. Without training as a physician, it is expected to be difficult to work in remote areas without special training [19]. It can be said that gender disadvantage also exists in the medical profession.

We assumed that gender and years of experience (years in practice after obtaining a medical license) are the most important factors influencing the distribution of physicians. We decided to study these two factors by post-licensure (years of experience) and by gender, using individual physician data.

## Methods

We studied the three time points of 1994, 2004, and 2014 using e-stat, which is distributed as access-free open data provided by the Statistics Bureau of the Ministry of Internal Affairs and Communications [20]. Population indices were taken from the Japanese national census conducted every five years. A database of all physicians was available from the Ministry of Health, Labour and Welfare Open Data Source [21]. The survey of physicians, dentists, and pharmacists is a reliable data set because it is a biennial status report system mandated for physicians, dentists, and pharmacists (although certain omissions may occur for physicians who are not working), and the Japanese system is able to capture almost all the status of physicians working at medical facilities. Personal physician information, such as place of employment, age, gender, and number of years since being licensed, was obtained directly from the Ministry of Health, Labor, and Welfare. The population of the municipalities was adjusted to the size of the most recent census in 2014 (1,741 municipalities), although there were some municipalities with zero population in the 2015 census due to the Great East Japan Earthquake in 2011. Since the census year did not match the physician survey year, we used the most recent year combination (1994–1995, 2004–2005, and 2014–2015) as the physician survey-census combination).

We conducted a detailed examination of the changes in the number of physicians over the 20-year period from 1994 to 2014, with an emphasis on changes by gender and years of experience. The initial classification of years of experience was by five-year intervals, but, since our previous studies [14] have shown that the distribution of young physicians fluctuates greatly within the first 10 years of licensure, the survey was subdivided into smaller categories for physicians with less than 10 years of experience.

We hypothesized that five factors could significantly affect physician distribution: number of physicians to population, number of physicians, municipal population, distance (from the prefectural capital), and population density. We combined two of each of these five factors and calculated correlation coefficients using the Spearman correlation. Correlation coefficients were analyzed at three time points (1994, 2004, and 2014) for all physicians, male physicians, and female physicians. A total of 24 correlation coefficients were obtained.

We surveyed 47 prefectures in Japan (*N* = 47) at three time points to determine how many physicians are distributed in the most populous one-third of each prefecture (urban areas), divided into overall physicians, male physicians, and female physicians. Our focus on the “one-third” of the population is based on the National Statement of Depopulation Measures 2019 [22]. This document defined isolated areas, population and the number of municipalities. "The remote areas occupy about 60% of Japan's land area" in definition, Our study set the top 1/3 as urban areas.

Finally, Gini coefficients for the three time points were obtained based on gender and years of experience. The number of physicians per 100,000 population was calculated for 1,741 municipalities using the population of each municipality listed in the national survey as the denominator. The Lorenz curves were sorted by the number of physicians per 100,000 population, with the cumulative municipal population (cumulative relative population) on the x-axis and the cumulative number of physicians in the medical profession (cumulative relative number of medical personnel) on the y-axis; the Gini coefficients were compared. The Lorenz curve is a commonly used method of analysis for social imbalance and can be visually understood by density on the x- and y-axes from the lower to the upper population groups [23]. The Gini coefficient is an integral value-based indicator derived from the Lorenz curve. It has the advantage of providing a visual representation of the Lorenz curve: if the Gini coefficient is close to 0, the Lorenz curve is in line with the ideal line (equal distribution), and if the Gini coefficient is close to 1, the Lorenz curve deviates from the ideal line (skewed distribution) [24]. In our previous study, we calculated the Gini coefficients for all physicians at three time points (1994, 2004, 2014); in this study, we calculated the Gini coefficients for male and female physicians based on years of medical experience after obtaining their licenses. This is the first attempt in Japan to investigate the distribution by gender and years of experience using individual physician questionnaires. Lorenz curves were plotted by gender and by years of post-licensure experience, and changes in Gini coefficients were determined over a 20-year period. Based on these results, we discussed the factors that have a significant influence on the distribution of physicians.

The Gini coefficient is an analytical method originally used for income distribution studies, but it had also been applied to the evaluation of physician distribution in Japan and other countries as well. We have also used the Gini coefficient to evaluate the distribution of physicians many times in previous studies [7, 9, 13, 14, 25–28]. We also have experience with analyses using individual physician information [10], which has the advantage of facilitating comparisons with these previous studies.

## Results

**Fig. 1:**
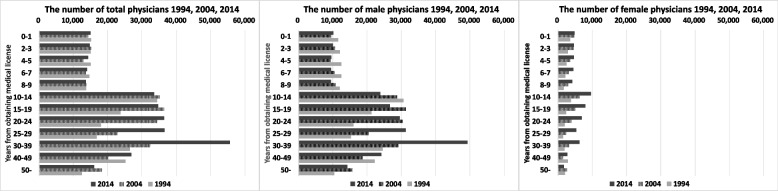
The number of physicians change in 1994–2014 by gender and years of experience

Figure 1 shows the change in the actual number of physicians 1994–2004-2014. From left to right, the changes over time are shown for all physicians, male physicians, and female physicians. The vertical axis represents the number of years since obtaining a medical license (years of medical experience), and the horizontal axis represents the number of physicians (persons).

Using 1994 as a baseline, the number of physicians as a whole has increased by a factor of 1.29 over the past 20 years. Female physicians increased by a factor of 2.17, and the ratio of female physicians to the total has increased. Male physicians increased by a factor of 1.23, but the number of male physicians up to year 14 decreased, indicating that only male physicians in years 15–19 contributed to the increase in male physicians. In other words, the ratio of female physicians is increasing by that much, and on the flip side, the number of veteran male physicians is expected to decrease rapidly.

Table 2 shows the correlation coefficients of the five indices for total physicians, male physicians, and female physicians, respectively. Since our analysis of Gini coefficient was based on the number of physicians to population ratio, Table 2 is also based on the number of physicians to population ratio and the actual number of physicians. Physicians to population, municipality population, and number of physicians, population density were in ascending order, but only the distance from prefectural capital indicator was in descending order (sorted by proximity to the prefectural capital).

**Table 2:** Spearman correlation coefficients for the 5 factors (assumed to influence physician distribution)

**Total Physician**		**1994**	**2004**	**2014**
Physicians to population (per 100,000)	Municipal population	0.511	0.529	0.566
	Number of physicians	0.745	0.752	0.772
	Distance from prefectural capital	0.194	0.222	0.270
	Population density	0.278	0.312	0.376
Number of physicians	Municipal population	0.943	0.948	0.952
	Distance from prefectural capital	0.436	0.460	0.484
	Population density	0.626	0.658	0.697
**Male Physician**		**1994**	**2004**	**2014**
Physicians to population (per 100,000)	Municipal population	0.483	0.498	0.526
	Number of physicians	0.728	0.730	0.744
	Distance from prefectural capital	0.160	0.177	0.222
	Population density	0.238	0.264	0.312
Number of physicians	Municipal population	0.940	0.947	0.950
	Distance from prefectural capital	0.426	0.449	0.472
	Population density	0.616	0.647	0.686
**Female Physician**		**1994**	**2004**	**2014**
Physicians to population (per 100,000)	Municipal population	0.603	0.615	0.674
	Number of physicians	0.870	0.869	0.897
	Distance from prefectural capital	0.397	0.410	0.440
	Population density	0.501	0.508	0.573
Number of physicians	Municipal population	0.888	0.900	0.910
	Distance from prefectural capital	0.470	0.490	0.509
	Population density	0.647	0.672	0.709
**(Supplementary correlation value)**		**1994**	**2004**	**2014**
Population density	Municipal population	0.687	0.714	0.746
	Distance from prefectural capital	0.654	0.658	0.654
Municipal population	Distance from prefectural capital	0.477	0.495	0.509

Physicians to population (per 100,000 population) / municipal population showed only some correlation 0.511–0.529–0.566 (1994–2004-2014), but the physicians to population / municipal population correlation was the highest at 0.943–0.948–0.952 (1994–2004-2014). We had expected a correlation with population density, but the correlation value for physicians to population/population density was not very high, 0.278–0.312–0.376. We had also planned to calculate the Gini coefficient with population density, but the low correlation led us to discontinue the analysis. Distance (from the county seat), a new indicator in this study, had a low correlation value. The correlation values were low, 0.194–0.222–0.270 (physicians to population / distance), 0.436–0.460–0.484 (number of physicians / distance). Correlation coefficients were also calculated for municipal population/distance, where physician distribution factors were not relevant, and were 0.477–0.495–0.501, showing some correlation, but lower than the population correlation. Looking at the respective correlation indices for male and female physicians, there were clear differences by gender. In terms of physician to population, municipal population, actual number of physicians, distance, and population density all have significantly higher correlation values than male physicians. All of the indicators picked up in this study were analyzed based on the expectation that they would correlate with the distribution of physicians, but it was unexpected that there would be such a difference between male and female physicians. In summary, as can be said for both men and women, the correlation between physician distribution and each indicator had mostly increased over time. In other words, looking at geographical factors, there is a strong possibility that physicians of both sexes are increasingly choosing (or "being in an environment where they can choose") to work in urban areas or in places with good transportation conditions.

We surveyed 47 prefectures to determine how many physicians are distributed in the top one-third of the population centers. The results are shown in Table 3.

**Table 3:** Population and physician ratio of the top 1/3 municipalities in Japanese 47 prefectures

(%)	1994			2004			2014		
	Max	Median	Min	Max	Median	Min	Max	Median	Min
Population	87.6	77.7	62.2	88.9	78.9	58.9	90.2	79.2	64.2
Total physician	95.5	86.0	52.0	96.1	86.7	54.2	96.9	87.7	52.8
Male physician	95.3	86.1	52.4	95.8	86.6	55.3	96.6	86.8	55.0
Female physician	97.4	88.8	48.3	98.6	89.0	48.7	98.9	91.8	43.7

We found that generally about 80% of the population in these municipalities. This was similar to the remote/urban population ratio in [22] introduced in Methods. However, there were prefectures where the population was concentrated in one area due to geographical factors, etc., and conversely, there were prefectures where the distribution was not uneven. In addition, there were outliers with extremely low minimum values for both male and female physicians. These were the case of medical school hospital located in a small municipality. Based on the above, we considered that the median value would be less susceptible to extreme outliers in determining the percentage of population and physician distribution. In some prefectures, the distribution of physicians was extremely concentrated in the top municipality (urban area) in terms of population. Hokkaido and Nagano prefectures, which have a large area, municipalities, and concentration of population in the center of the prefecture, were representative examples. Fukuoka and Tottori prefectures are also examples of prefectures with a small area but a large concentration of physicians. These were thought to be due in part to the geopolitical background of having few plains and a concentration of remote municipalities in mountainous areas.

Judging from the secular change in the median, 77.7–78.9–79.4% (median) of residents distributed in the top one-third of the population centers over the course of each decade. Paralleling this population trend, 86.0–86.7–87.7% (median) of physicians were distributed in the top one-third most populous areas. In the municipality with the highest distribution rate, 96.9% of physicians were concentrated in these areas as of 2014, highlighting the reality that the increased concentration of population in urban areas and the concentration of physicians are occurring simultaneously. In addition, 88.8–89.0–91.8% (median) of female physicians were concentrated in the most highly populated municipalities, which was a higher distributional concentration compared to male physicians.

**Fig. 2:**
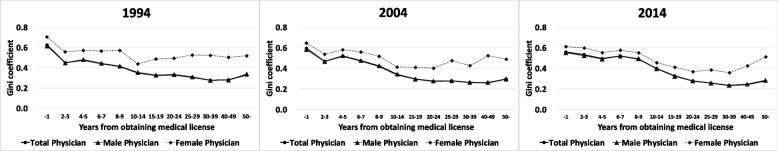
Gini coefficient transition for Japanese physicians:1994, 2004, and 2014

In this study, 12 Lorenz curves were created for total physicians, male, and female, classified by years of physician experience. A total of 36 Lorenz curves were created, and Gini coefficients were calculated for each. Figure 2 summarized the above results, plotting years of experience on the x-axis and corresponding Gini coefficients on the y-axis, connected by a fold line. The changes over the years of experience for physicians as total, male, and female physicians, respectively, can be seen briefly. The Gini coefficient graphs for the three time points studied (1994–2004-2014) are side-by-side.

The Gini coefficients for all physicians varied from 0.315–0.298–0.298. The Gini coefficient of total physicians improved between 1994 and 2004, but the Gini coefficient did not change between 2004 and 2014, i.e., the result of a less advanced distribution to remote areas in terms of population factors. The Gini coefficient for male physicians steadily decreased from 0.311–0.289–0.283 (male physicians distribution to remote areas is progressing), while the Gini coefficient for female physicians did not decrease from 0.394–0.385–0.395 and the Gini coefficient has remained high for the past 20 years (they have remained in urban areas and have not been distributed to more remote areas). Looking at post-licensure, the Gini coefficient had decreased for both male and female physicians, especially among mid-career physicians in their 10th year after graduation. However, even during that period, the Gini coefficient for female physicians was higher than that of male physicians, and female physicians had a lower tendency to be distributed in remote areas than male physicians at any age. In other words, this suggests the current situation in which veteran male physicians are the core of remote area medicine in Japan. In the 1994 and 2004 surveys, the Gini coefficients were much lower for the group of physicians with 2–3 years post-graduation, but there was no significant decrease in 2014. This is indicative of the impact of the start of the new clinical training system on the distribution of physicians.

## Discussion

We studied the urban and rural distribution of physicians, examining whether they were male or female and younger or more experienced. The results showed significant differences in urban/rural distribution trends between male and female physicians and between experienced physicians and those who were more recent medical school graduates.

The results of Fig. 1 indicate that it is imperative to respond to the increasing number of female physicians in healthcare policy. Because the government has an obligation to enforce medical policies that can accommodate the life events of female physicians, and provide a stable supply of medical care to the public as well. Including the facts in Table 1, the number of female physicians will account for about 30% in the near future, and may even reach 50% in the distant future.

For the discussion of Table 2, physicians are distributed in ascending order of population, and furthermore, this correlation value had increased over time. This suggested that physicians may be more likely to be distributed in areas with large municipal populations.

The lower correlation value for physicians to population ratio is also evidence of the existence of an imbalance in physician distribution. Densely populated areas are more likely to have general hospitals with many specialties, and general practitioners (clinics) with many departments are more likely to congregate. On the other hand, there are many departments that do not exist in remote areas (obstetrics and gynecology, dermatology, ophthalmology, etc. are particularly prominent), and the reality is that medical and surgical specialists of each department are responsible for regional medical care as generalists. This is a form of medical care that can be seen throughout Japan, and the above factors must be taken into consideration when the correlation values between physicians to population/municipality, and the number of physicians/municipality differ significantly.

Although the correlation between distance and physician distribution was stated to be lower than the correlation between population and physician distribution, the correlation value has increased over time. Furthermore, the correlation of municipal population/distance, which was unrelated to the correlation of physician distribution, was increasing. It indicates that population is generally concentrated in municipalities that are closer to urban areas. The fact that physician distribution is correlated with population distribution (especially for female physicians, who are increasing in number), the increase in the correlation of distance cannot be ignored. The distance factor may also suggest that the distribution of physicians is likely to slow down in the future.

Further, considering the results in Table 3, it is necessary to understand why many female doctors choose urban areas rather than remote areas when they go into practice. Previous studies have reported that female physicians struggle with work–life balance [29–31]. Since women must balance their careers with life events such as marriage, pregnancy, childbirth, and childcare, it is understandable that women would choose to work in urban areas where it is easier to take leave (e.g., general hospitals where it is easier to find a substitute doctor), work part-time, or work shorter hours. Urban areas also provide more career choices for those wishing to pursue a medical specialty or other paths in the medical field.

As discussed in Fig. 2, the Gini coefficient for physicians to population ratio has generally declined over time, suggested that physicians were distributed in more remote areas. In other words, it was likely that the results would be different if the Gini coefficients were calculated using the actual number of physicians sorted. However, our and the other previous studies presented in "Background" had also compared Gini coefficients using the physician population ratio sort. If the number of physicians sorting is used, the outliers are likely to be larger in municipalities with large general hospitals or university hospitals in small-population municipalities, as mentioned earlier. In our experience, we thought that the Gini coefficient comparison with the physicians to population ratio sort is more representative of the actual situation regarding physician distribution.

The 2014 analysis showed a large distribution discrepancy between the younger group of physicians compared to those who had been licensed for 30 years or more, with the two groups having Gini coefficients of 0.560 and 0.234, respectively. The 2014 coefficients for male and female physicians were 0.283 and 0.395, respectively, showing a large discrepancy between the genders. While the distribution of male physicians paralleled overall trends, female physicians had higher Gini coefficients, and even veteran female physicians had a lower distribution to rural areas. While there were higher numbers of veteran physicians of both genders in rural areas, gender differences remained Fig. 2, and experienced male physicians provided much of the care in those areas.

These findings show the need for future research on female physicians who wish to have a career while also choosing to have children and a family life. Of course, these choices affect the woman’s partner, who will also remain in the more populated areas. Men can contribute to their partners’ work–life balance by assisting in childcare and housework duties. Experienced medical professionals, like the authors of this paper, know the clinical field well and can provide advice to their younger colleagues.

Based on several reports [32–35], it is clear that all physicians are suffering from considerable physical and mental fatigue, both in Japan and globally, and that female physicians, in particular, are struggling to balance their careers with housework and child-rearing. A 2006 survey report on the employment rate of physicians reveals that while 90% of Japanese male physicians were employed, the rate for female physicians gradually declined after obtaining a medical license, and a quarter of female physicians were not working by the 10th year [36].

This trend was similar for women of child-bearing age in all occupations who were balancing the multiple demands in their lives. Childbirth (70.0%) and childcare (38.3%) were the main reasons for interrupting or leaving work [37]. Japanese physicians are free to choose their specialty after completing clinical training, and a 2012 Japanese survey showed that the specialties with the highest percentages of female physicians were dermatology (44.3%), ophthalmology (37.5%), and anesthesiology (36.3%) [36]. It is assumed that women will choose workplace environments that are conducive to the needs of female physicians and where it is easier for them to voice their concerns about work–life balance and find solutions.

In a survey of female physicians, 25.9% said understanding and atmosphere in the workplace were the number one conditions for being able to continue working while raising children [31]. Female physicians also tended to choose pediatrics (33.7%) and obstetrics/gynecology (31.5%), specialties that parallel their traditional roles in society. The least popular specialties for female physicians were surgery (7.1%), urology (5.0%), and orthopedics (4.4%) [36], indicating that female physicians are less likely to choose specialties that are strenuous, require a lot of overtime. An increase in the percentage of female physicians will not have a significant impact on the overall number of physicians, but the number of practitioners is expected to decrease significantly in specialties such as surgery, where the participation rate of female physicians is very low.

We believe the increasing numbers of female physicians should be supported so they can continue their career steps (including securing clinical and research posts when their careers are interrupted by life events). If the sustainability of rural hospitals is to be maintained, the challenge is to create an environment supporting a work–life balance that allows female physicians to work steadily while building their careers.

One of our proposals for a specific healthcare policy is to expand the system of general practitioners. The definition of “general practitioner” and the training process for general practitioners still need to be improved. However, female physicians who choose to become generalists can respond to the demand in rural areas while also raising a family [38, 39]. However, it is also necessary to study how many female doctors might choose to become general practitioners.

This analysis also allowed us to visually assess the impact of the clinical training system in Japan. Most hospitals registered as clinical training institutes are university-affiliated hospitals and general hospitals located in urban areas, where there is more case experience, and these attract more trainees than corresponding regional core hospitals in rural areas.

In the past, most physicians within two years of graduation were assigned to university hospital departments after short-term training at medical school and were dispatched to regional medical institutions through personnel. However, physicians themselves are now able to freely choose their post-residency careers, which will further decrease the extent to which they choose to practice in rural areas. There is also a growing number of fields, such as preventative medicine and public health, that are available for physicians to choose from, and this trend should be further investigated.

The clinical training system for physicians has succeeded in removing the negative aspects of the past segmentation into specialties, broadening the scope and insight of individual physicians, and creating physicians who are capable of providing comprehensive care, resulting in improved access to medical care. However, when the policies that were thought to be right on a micro level are combined with the current situation in Japan, they have resulted in negative outcomes that could not have been predicted on a macro level, such as a shortage of doctors in rural areas, a concentration of young physicians in urban areas, and the fact that veterans are supporting local rural medical services.

This study was based on a comparison of the entire physician population at three time points. However, because it focused on "male and female physicians" and "younger and veteran physicians" at each time point, the other comparators (symmetry) were not clear. The database used in this study, individual physician data, also allowed comparison of "department," "outpatient and inpatient care," "ratio of physicians engaged by hospital size," etc. However, if these data were included, the main focus of the paper would be on "male and female physicians" and "young and experienced physicians. However, since the inclusion of these data would blur the main findings of the paper, we considered them to be a limitation of the research approach used in this study and would like to address them in a future study.

## Conclusion

Medical care in rural areas in Japan has become dependent on male physicians who have been in the field for many years. A large percentage of young physicians, and an even larger percentage of female physicians, choose to remain in urban areas. Many women choose urban areas because it is easier to sustain a work–life balance in practices where they can easily take time away from work to meet family demands. To sustain adequate healthcare for the population of rural Japan, it is necessary to create conditions in those regions that allow female physicians to develop their careers while achieving a work–life balance.

### Supplementary Information

**Additional file 1.**

## Data Availability

The data that support the findings of this study are available from the corresponding author, Kazuki Kimura, upon reasonable request.

## Abbreviation

OECD: Organization for Economic Co-operation and Development
