# Supplementary figures and images for: Gender difference of geographic distribution of physicians in Japan: three-point analysis of 1994, 2004 and 2014
Source: BMC Health Serv Res. 2023 Dec 13;23:1404. doi: 10.1186/s12913-023-10258-4 (PMC10720184; doi:10.1186/s12913-023-10258-4)

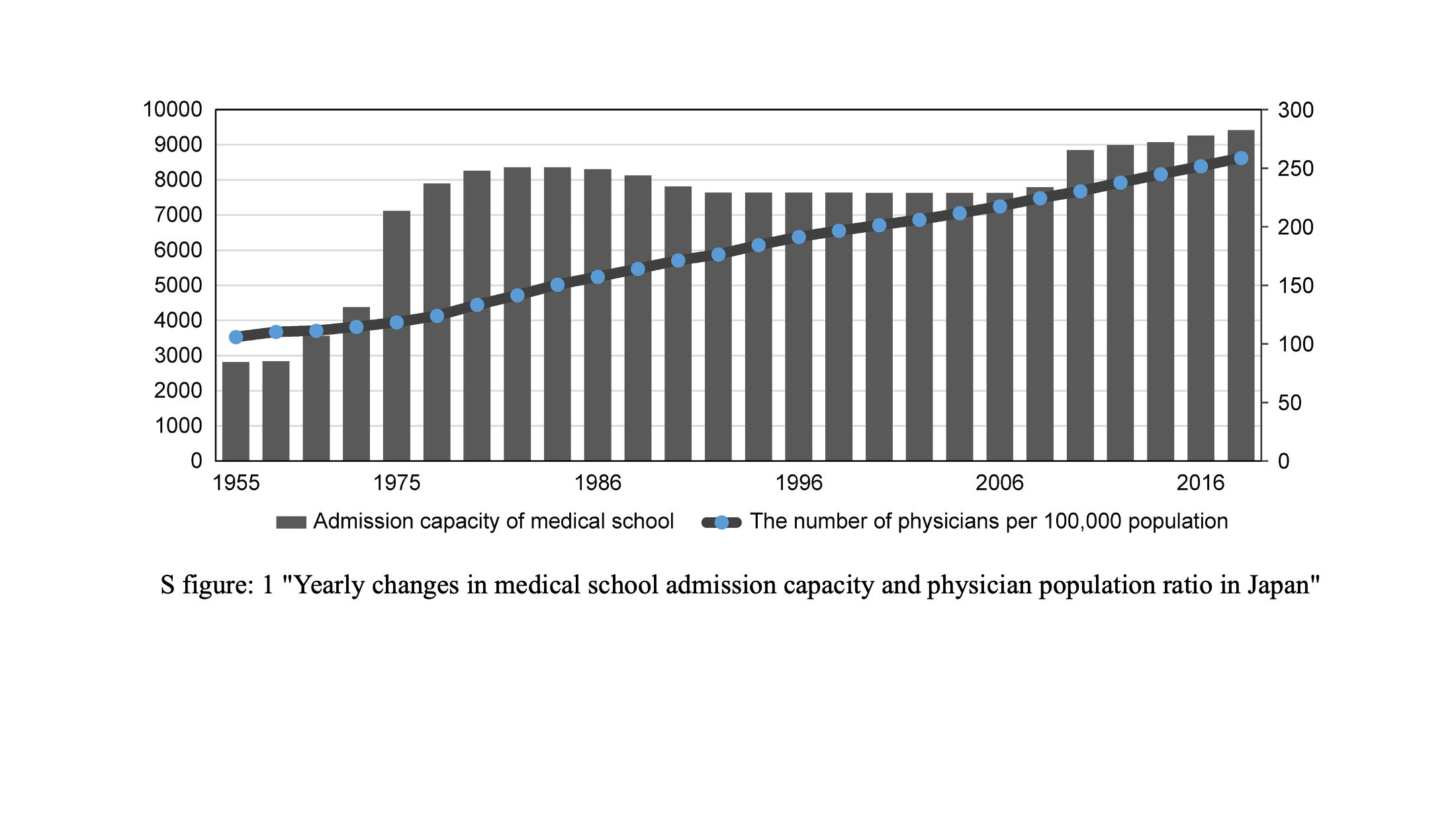

Supplement: Supplementary file 1 — Additional file 1. [file 12913_2023_10258_MOESM1_ESM.tiff]
